# A Strange Conditional Legacy

**Published:** 1920-09-11

**Authors:** 


### A Strange Conditional Legacy.

Mr. Ernest Leopold Walford, a gentleman who was well known to a number of hospital secretaries in London, and one who formerly attended many festival dinners, died recently after a very short illness. In his will he instructed his executors to arrange that immediately after his death one of the principal arteries in his body shall be opened by a qualified surgeon and a proper fee paid to him. Should this act be carried out he left the whole of his property to the Jews' Hospital and Orphan Asylum, to create annuities. Subject to this condition Mr. Walford left a sum to be invested and the interest to be used for the servants' Christmas fund of his favourite club, and another fund to the Jewish Board of Guardians for annuities for widows. An eighth part of the residue is left to the Jews' Hospital and Orphan Home for two fresh annuities. At one time Mr. Walford, who was a bachelor, lived near a well-known lying-in hospital, and he was so frequently favoured by visits from would-be patients, who had ascertained his name with a view of obtaining a "letter," that the calls upon him in this respect became an embarrassment, and he had to ask the secretary to remove his address from the list of subscribers.

